# Virtual screening of a MDR-TB WhiB6 target identified by gene expression profiling

**DOI:** 10.6026/97320630015557

**Published:** 2019-09-05

**Authors:** Mahalakshmi Vijayaraj, PA Abhinand, PK Ragunath

**Affiliations:** 1 Department of Bioinformatics, Faculty of Biomedical Sciences, Sri Ramachandra Institute of Higher Education and Research (Deemed to Be University) Porur, Chennai-600116

## Abstract

Multidrug resistance in M. tb has become a huge global problem due to drug resistance. Hence, the treatment remains a challenge, even though short term chemotherapy is available. Therefore, it is of interest to identify novel drug targets in M.tb through gene expression profiling complimented by a subtractive proteome model. WhiB6 is a transcriptional regulator protein and a known drug resistant marker that is critical in the secretion dependent regulation of ESX-1, which is specialized for the deployment of host membrane-targeting proteins. The WhiB6 protein structure was modelled ab initio and was docked with a library of 173 phytochemicals with potential antituberculosis activity to the identified drug marker to find novel lead molecules. UDP-galactopyranose and GDP-L-galactose were identified to be potential lead molecules to inhibit the target WhiB6. The results were compared with the first line drugs for MDR-TB by docking with WhiB6. Data showed that Ethambutol showed better binding ability to WhiB6 but the afore mentioned top ranked phytochemicals were found to be better candidate molecules. The chosen candidate lead molecules should be further validated by suitable in vitro or in vivo investigation.

## Background

Every year about 10 million people are affected by tuberculosis and among which 1.6 million people die. [1-2] Across the world about 10 million people developed tuberculosis as of 2017 about two third of all new cases occurred in 8 countries like India, China, Indonesia, Philippines, Pakistan, Nigeria, Bangladesh and South Africa which are designated the status of high TB burden countries along with 22 other countries. These countries contribute to 87% of world cases.[1] Multidrug resistance in Mycobacterium tuberculosis has emerged as a major problem in treatment even though short term chemotherapy is available; development of resistance to antibiotics has become a global menace. [3] MDR-TB does not acquire drug resistance due to transposable element or a plasmid carrying drug resistant marker, but instead it is acquired by stepwise new mutations in genes for different drug targets. [4] Resistance against the major first line antituberculosis drugs - treptomycin, Ethambutol, Pyrazinamide, Isoniazid and Rifampicin makes it necessary for treatment with second line drugs with greater toxicity and lesser efficacy. [5] Exuding antibiotic is due to the impermeable cell wall, that is mediated by efflux mechanisms by several ABC (ATP - binding cassette) transporter and major facilitator super family (MFS) proteins. Among the other causes for drug resistance, efflux mechanism contributes in a major way to intrinsic resistance to drugs. [6] Currently the growing trends of drug resistance in M.tb have led to a wide range of drug discoveries and to look for the functional protein that which is of key focus to target a lead molecule. In this scenario alternate treatment protocols with lesser toxicity can help clinicians battle MDR TB with greater ease. In the current study we have attempted to recognize novel drug target in M.tb through gene expression profiling approach complimented by a subtractive proteomic approach. Subsequently a library of Phytochemicals with potential antituberculosis activity, virtual screening was performed against the identified biomarkers to find novel lead molecules to combat MDR TB (Figure 4).

## Methodology

### Systematic search for gene expression datasets pertaining to MDR-TB:

A comprehensive literature mining of all eligible studies on Mycobacterium tuberculosis gene expression was carried out by searching GEO datasets (as on December 2016) based on the search terms X1 AND (("I" OR "i" AND (T OR t)) X2 AND (("I" OR "i" AND (T OR t)) Where, X1 = Gene expression; X2 = Microarray; I =Mycobacterium tuberculosis; i= Mtb; T = Tuberculosis; t = tb The concept concordance was limited to Tuberculosis, so that only datasets containing studies or data related to TB would be pulled out. Further the confidence of mining was tested by simple scoring algorithm. (Shown in Table: 1) Out of these only those gene expression datasets pertaining to Multidrug resistant tuberculosis strains and /or clinical isolates were considered for analysis.

### Gene expression profiling:

Gene expression profiling is a technique aimed at understanding transcription pattern in a cell at a given time frame. Measuring mRNA levels is accomplished by measuring mRNA levels of individual genes. Usually relative mRNA levels in two or more experimental conditions (case Vs control) are measured to analyze and understand specific gene expression pattern in given condition Pre-processed datasets were chosen by systematic text mining technique as described above. [7]Based on the systematic literature search as described above, microarray datasets were retrieved from NCBI.GEO repository https://www.ncbi.nlm.nih.gov/gds/?term= mycobacterium+tuberculosis) using accession number GSE3201 annotated in GPL2787 platform which provides complete coverage of the Human Genome (Build 133, April 20, 2001) plus 6500 additional genes for analysis of over 47,000 transcripts. Gene expression profiling analysis of the chosen dataset using GEO2R. [8] The dataset comprised of gene expression data from 11clinical isolates and H37Rv as the (reference strain) as control. Each of the 11 clinical isolates was compared against H37Rv individually by using GEO2R log transformation was applied to all the data prior to analysis. Bonferroni adjustment was applied to the p-values. In each of the 11 comparisons, only those genes which showed log fold change >1.5 was taken for the further analysis (depicted in table). The upregulated genes which were common in all the clinical isolates (while comparing them with H37Rv) were chosen as candidate drug targets. The genes- MmpL10, WhiB6, Rv1052, PPE39, and Rv2035 were found to be upregulated in all the isolates. From these 5 genes WhiB6 was chosen as the suitable candidate drug target based upon several filtering parameters discussed in detail in the results and discussion section.

### Protein Modelling:

Determination of protein 3D structure is an essential part of many aspects of molecular research. In the absence of an experimentally determined protein structure (from X-diffraction or NMR) computational prediction of protein 3D structure becomes the only alternative. Computational protein structure prediction is highly beneficial in gaining insights on the protein function and drugs screening. [9]

### Ab-initio Modelling:

The primary sequence of WhiB6 from H37Rv retrieved from UniprotKB ID No P9WF37. The protein sequence was subjected to a PSI Blast against PDB database to recognize suitable template for modelling WhiB6 by homology method. Due the absence of any structurally similar orthologs with a solved structure, Ab-initio modelling was chosen. Ab-initio protein structural modelling is employed when the protein of interest does not have any homologue with solved structure to be used as template for modelling. Ab-initio modelling performs a conformational scan based on designed energy function. QUARK is a computer algorithm for Ab initio protein structure prediction and protein peptide folding, which constructs the correct protein 3D model from small fragments, by replica exchange Monte Carlo simulation under the guidance of an atomic level knowledge- based force field. It conducts a conformational search of a designated energy function, which enables to generate a number of possible suitable structures. [10] The sequence was subjected to PSI-Blast against the human genome to rule out the presence of human orthologs with high sequence similarity.

### Model validation:

The model obtained by Ab-initio modelling using Ramachandran plot, ERRAT2 and ProSA. Ramachandran plot was obtained from the Pdbsum server.

### Library of Phytochemicals- used as potential lead molecule against tuberculosis:

Phytochemical were searched for using systematic literature search. Only those compounds with pro1 antituberculosis activity were chosen and their 3D structures in Dot Sdf format were taken. Those Phytochemicals which did not abide by Lipinski's rule of 5 were filtered out and rest of the compound was taken for further analysis.[11-15]

### Molecular docking:

The library of Phytochemicals with reported antituberculosis activity subjected to virtual screening against WhiB6 (H37Rv) using Molegro virtual docker. Molegro Virtual Docker (MVD) 5.0 uses MolDock scoring system and it is based on a hybrid search algorithm, called guided differential evolution. This algorithm combines the technique of differential evolution optimization with a cavity prediction algorithm. The modelled protein structure was loaded on to MVD 5.0 platform for the molecular docking process. The built-in cavity detection algorithm of MVD 5.0 was used to identify the potential binding sites which are also referred to as active sites or cavities. The search algorithm used was MolDock SE and 10 was the number of runs taken while 2000 was the maximum iterations for a population size of 50 having 100 as the energy threshold. At every step, least 'min' torsions/translations/rotations were sought and the molecule having the lowest energy was preferred. After molecular docking simulation, the poses (binding modes) obtained were classified by re-rank score. Using the ligand preparation module of MVD 5.0, the selected ligands were manually prepared. Bond order, flexible torsion and the ligands were deducted. After the careful removal of hetero atoms and water molecules, the target protein structures were prepared and its electrostatic surface was produced. The grid resolution was set at 0.3 Å. The maximum interaction and maximum population size were set at 1500 and 50 respectively. Further the first line MDR-TB drugs- Ethambutol, Streptomycin, Pyrazinamide, Isoniazid, Rifampicin were docked against WhiB6 to measure the relative affinity and mode of interaction of these first-line drugs in comparison with the Phytochemicals which were found to posses the best binding affinity towards WhiB6.

## Results and Discussion:

### Gene expression profiling

Gene expression profiling of the 11 clinical isolates was performed using GEO2R by comparing each of the isolates against H37Rv (taken as control). Bonferroni correction was applied to the p-values to counteract the problem of multiple comparisons. Those genes that were at least 1.5 fold upregulated in each of these clinical isolates were tabulated and were shown in Table 2. The genes- MmpL10, WhiB6, Rv1052, PPE39, and Rv2035 were found to be upregulated in all the isolates. Amongst these 5 genes Rv1052 and Rv2035 were uncharacterized proteins and thereby were not included in the further analysis. PPE39 has number of genetic variance across, the different M.tb isolates caused by SNPs or 1S6110 integration. Owing to the high degree of variability PPE39 was not considered to be a suitable drug target. [16-17] MmpL10 (Rv1183) translocates diacyltrehaloses (DAT) across the plasma membrane where they are further acylated to generate pentacyltrehaloses (PAT). Still the role of MmpL10 in the virulence of mycobacterium tuberculosis is still unclear. [18-19] several studies on mice aerosol models revealed. DAT/PAT deficient M.tb was more virulent and infected macrophages readily. Based on the functional redundancy and a 'little' importance in the virulence process, MmpL10 might not be an ideal drug target. [19-21] Further more MmpL10 was a large protein (1006 amino acid long) and lacked structure solved homologues. This was revealed by performing a PSI-Blast of MmpL10 against the PDB database. Therefore, MmpL10 is not be modeled by homology method.

WhiB6 is critical in the secretion dependent regulation of ESX-1 substrate which one of the secretion system that is deployed to target host membrane targeting protein. It is responsible for the secretion of ESAT-6 which is one of the most major and well studied virulence factors in M.tb. [22] ESX-1is involved in the transformation of a number of virulence factors. Perturbations in the ESX-1 gene cluster affects virulence and pathogenicity of M.tb drastically. [23]

### Modelling of WhiB6 and Target validation by subtractive proteomic approach:

**Figure 1:**
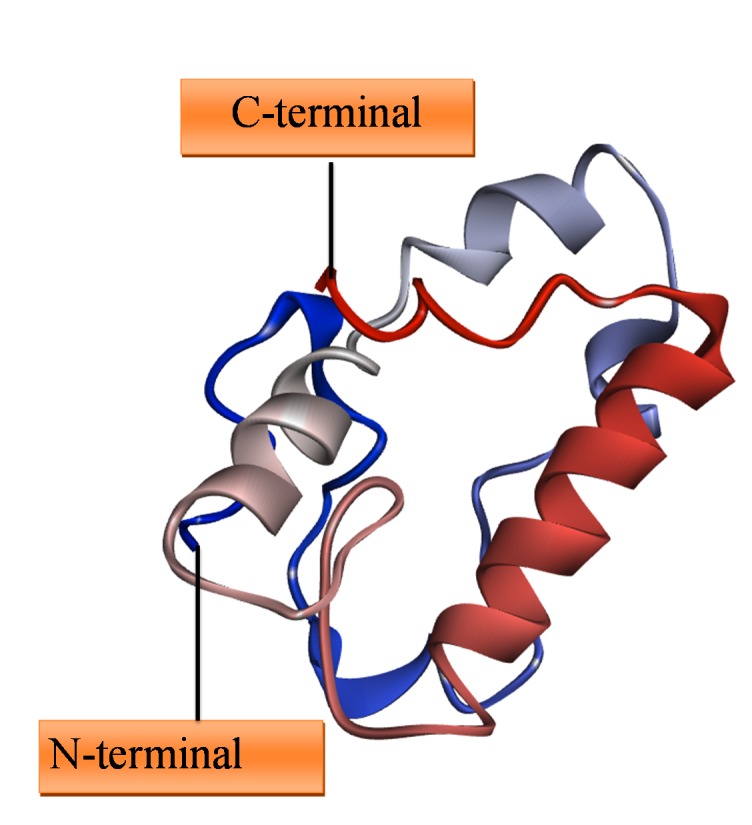
3D structure of WhiB6

PSI-Blast was performed to predict the suitable template with solved 3D structure to model the WhiB6 (H37Rv), this revealed that no structural orthologs with more than 40% of sequence similarity with WhiB6. Therefore homology modelling could not be employed for structure prediction of WhiB6, so Ab-initio modelling was employed as an alternative. WhiB6 protein was modeled by Ab initio modelling method by using QUARK server by taking small fragments through replica exchange Monte Carlo simulation method utilizing atomic level knowledge based force field. The built protein model was validated using Ramachandran plot to evaluate the stereochemicals stability of the modelled WhiB6. Ramachandran plot revealed that out of the total 101 non-glycine, non-proline residues present in WhiB6 -59 amino acids were present in the most favoured regions. 35 were present in the additionally allowed regions and further 5 amino acids were present in the generously allowed regions-totally constituting 98.0% of all residues. The number of amino acids in the disallowed regions was mere 2.01%. The presence of the vast majority of amino acids in the allowed regions of the plot shows that the modeled WhiB6 was stereochemically stable. [24] Errat2 server was employed to study the non-bonded interactions between the various atom types in the model protein. ProSA analysis revealed Z score of -5.69. Human protein shared more than 31% of similarity with H37Rv and WhiB6. It is generally hypothesized that protein sharing high degree of sequence similarity will also have structural similarity (Figure 1). Therefore lack of sequence and structural homologues in humans suggest that a lead molecule inhibiting M.tb WhiB6 will have very low propensity to cross bind with human Whib6 leading to adverse effects.

### Virtual screening of phytochemical library against WhiB6:

**Figure 2:**
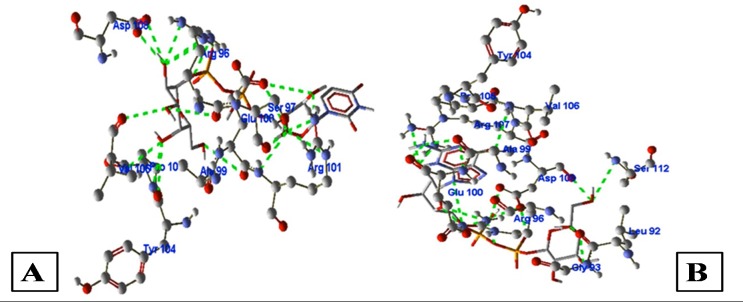
Illustration of docking poses of (A) UDP-galactopyranose interacting with WhiB6 (H37Rv), (B) GDP-L-galactose interacting with WhiB6 (H37Rv), the image depicts each ligand's interaction with the active site of WhiB6. The H-bonds are shown as green dotted lines, the ligand is shown in wire frame model and the protein in ball and stick model. CPK coloring scheme has been use.

A library of 173 Phytochemicals was subjected to virtual screening against WhiB6 of H37Rv using Molegro Virtual docker 5.0. Out of the 173 compounds the following 5 compounds: UDPgalactopyranose, Methoxy-hydroxyl-phosp GDP-4-Dehydro-6- deoxy-D-mannose, GDP-D-Rhamnose, GDP-L-galactose and Oceanapia were found to show highest binding affinity against binding cavity of WhiB6. The docked compounds were ranked on the basis of Molegro score, number of H-bonds and H-bonding energy. [25] (Figure 2)

UDP-galactopyranose binds with WhiB6 by forming nine H-bonds interacting with Glu100, Arg101, Ser97, Arg96, Ala99, Pro105, Pyr104, Val106, Asp108 with a MolDock score of -97.67 and H-bond of -20.06. Methoxy hydroxy phosp-GDP 4 Dehydro 6 deoxy D mannose binds with WhiB6 by forming 10 H-bonds interacting with Arg101, Ala99, Ser97, Glu100, Gly103, Tyr104, Pro105, Arg107, Asp108, and Arg96 with a MolDock score of -105.49 and H-bond of -13.45. GDP D Rhamnose binds with WhiB6 by forming 7 H-bonds interacting with Asp108, Arg107, Val106, Pro105, Ala99, Glu100, and Arg96 with a MolDock score of -111.96 and H-bond of -12.83. GDP L galactose exhibited the highest binding affinity towards of WhiB6 as indicated by a high MolDock score of -115.80 and H-bond score -12.64. It formed a total of 11 H-bonds with binding cavity of WhiB6 interacting with the amino acids Tyr104, Pro105, Arg107, Val106, Ala99, Glu100, Asp108, Arg96, Ser112, Leu92, and Gly93. Oceanapia binds with WhiB6 by forming 7 H-bonds interacting with Gly103, Ala99, Glu100, Pro105, Arg96, Asp108, and Arg107 with a MolDock score of -105.27 and H-bond of -11.50 (shown in Table: 3).

**Figure 3:**
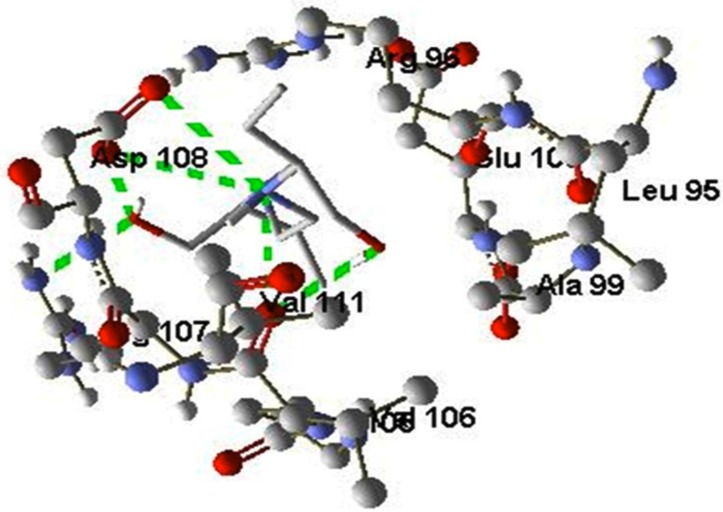
Illustration of docking poses of Ethambutol interacting with WhiB6 (H37Rv)

**Figure 4:**
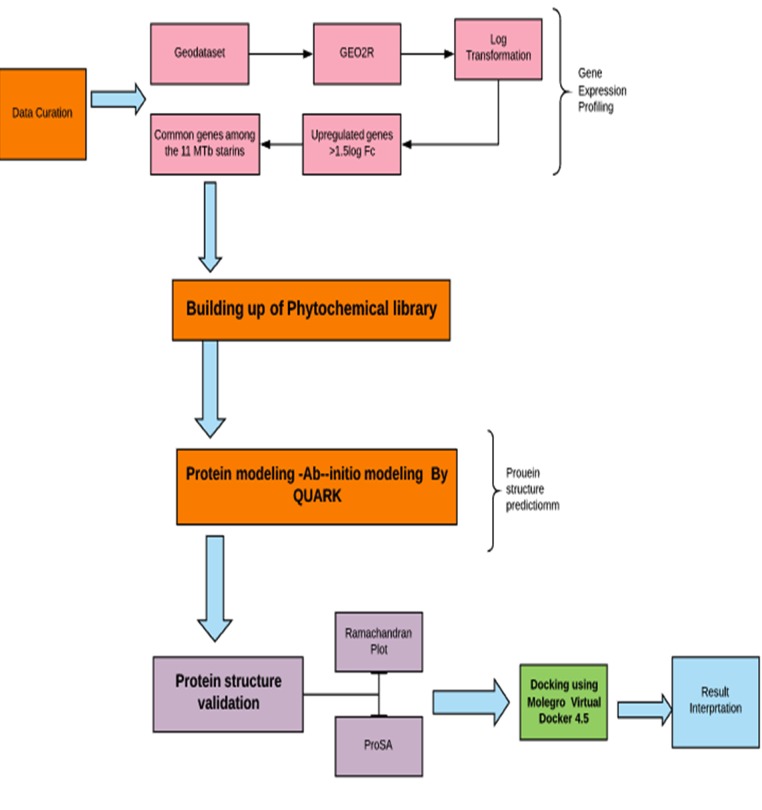
Flow chart illustrating the gene expression profiling, protein modeling and lead identification and Interpretation

UDP-galactopyranose belong to the class of Uridine Diphosphate Sugars commonly found in Cucurbit Fruit, Melons, and Legumes and GDP-L-galactose belong to the class of organophosphate oxoanion commonly found in tomato fruit, and strawberry are potential lead molecules against WhiB6 of M.tb based on their high binding affinity and the ability to form strong H-bonds. UDP-galactopyranose is further suitable as a lead molecule as it abides by all the Lipinski's rule of five. [11] Whereas GDP-L-galactose has a molecule weight of 605.34 and thereby might not be suitable for oral administration. The first line MDR-TB drugs were docked against WhiB6 to identify their potential WhiB6 inhibiting activity in comparison with the identified Phytochemical lead molecules. The molecular docking of Pyrazinamide, Isoniazid, Ethambutol, and Streptomycin against WhiB6 revealed that streptomycin and Rifampicin do not bind with WhiB6 as shown by a positive MolDock score 34.2929 for streptomycin and 967.456 for Rifampicin Table 4 . The H-bond score are 4.88673 and -5.15092 respectively. (Figure 3) Ethambutol showed the highest binding affinity towards WhiB6 compare to all the other first line MDR-TB drugs which is shown by a MolDock score of -78.1277 and it formed 6 H-bonds with amino acids-Asp108, Arg107, and Val111 but while comparing the binding affinity with top ranked Phytochemicals, the compounds such as UDP-galactopyranose, GDP-L-galactose showed much stronger binding affinity with WhiB6 and formed more H-bonds.

## Conclusion

WhiB6 is a transcriptional regulator protein, which is a known drug resistant associated marker in M.tb. It is an ideal candidate drug target to combat MDR-TB based on the results from gene expression profiling and subtractive proteomic approach. UDPgalactopyranose and GDP-L-galactose is the potential lead molecule to bind and inhibit WhiB6. The invitro and invivo efficacy of UDP-galactopyranose and GDP-L-galactose needs to be investigated further.

## Figures and Tables

**Table 1:** Systematic search for gene expression datasets pertaining to TB

S. No	Key words	Dataset size
1	Gene Expression AND (("Mycobacterium tuberculosis" OR " Mtb" AND (Tuberculosis OR tb))	1253
2	Microarray AND (( "Mycobacterium tuberculosis" OR " Mtb" AND (Tuberculosis OR tb))	548
3	Total	1801

**Table 2:** Phytochemical library of compounds with reported antituberculosis activity for virtual screening against Whib6

S. No	Phytochemicals Common Name	Compound CID	Biological activity
1.	Emivirine	CID:5366244	MDR TB
2.	Berberastine	CID 5785	MDR TB
3.	Phosphoglycolohydroxamic Acid	CID 442180	MDR TB
4.	Cinnamaldehyde	CID 2353	MDR TB
5.	Diallyl Disulfide	CID 637511	MDR TB
6.	Bilobalide	CID 16590	MDR TB
7.	Baicalin	CID 73581	Antituberculous
8.	3-Formylcarbazole (1)	CID 64982	Antituberculous
9.	3-Methoxycarbonylcarbazole (2)	CID:3091534	Antituberculous
10.	2-Hydroxy-3-Formyl-7-	CID:504069	Antituberculous
11.	Methoxycarbazole	CID 189687	Antituberculous
12.	Clauszoline J	CID 10797986	Antituberculous
13.	Echinuline	CID 504070	Antituberculous
14.	Pseudopteroxazole	CID 115252	Antituberculous
15.	Seco-Pseudopteroxazole	CID 6475529	Antituberculous
16.	Homopseudopteroxazole	CID 10614977	Antituberculous
17.	Flavonols	CID 3003592	Antituberculous
18.	Flavone	CID 11349	Antituberculous
19.	Dentatin	CID 10680	Antituberculous
20.	Nor-Dentatin	CID 342801	Antituberculous
21.	Methyl Clausenidin	CID 5495613	Antituberculous
22.	Chaetomanone	CID 5315947	Antituberculous
23.	Erogorgiaene	CID 5318998	Antituberculous
24.	7-Hydroxy Erogorgiaene	CID 9816893	Antituberculous
25.	Aureol N,N-Dimethyl-Thiocarbamate	CID 9816893	Antituberculous
26.	Potamogetonin	CID 5270653	Antituberculous
27.	Potamogetonyde	CID 5742898	Antituberculous
28.	Potamogetonol	CID 485584	Antituberculous
29.	(+)-Totarol	CID 485585	Antituberculous
30.	Secokauranes	CID 92783	Antituberculous
31.	Phorbol Ester	CID 101394720	Antituberculous
32.	Dustanin	CID 27924	Antituberculous
33.	15-Acetoxydustain	CID 12309402	Antituberculous
34.	Cycloartenol	CID 3010870	Antituberculous
35.	Stigmasta-4-En-3-One	CID 92110	Antituberculous
36.	Stigmasta-4,22-Dien-3-One	CID 5484202	Antituberculous
37.	B-Sitosterol	CID 6442194	Antituberculous
38.	Stigmasterol	CID 222284	Antituberculous
39.	Epidioxysterol	CID 5280794	Antituberculous
40.	Pregnene Saponin	CID 10789345	Antituberculous
41.	Jujubogenin Analog	CID 3010873	Antituberculous
42.	Physalin B	CID 15515703	Antituberculous
43.	Physalin D	CID 5488849	Antituberculous
44.	Preussomerin	CID 72551426	Antituberculous
45.	Deoxypreussomerin	CID 44332169	Antituberculous
46.	Punicalagin	CID 11078086	Antituberculous
47.	Hirsutellide	CID 16129869	Antituberculous
48.	Beauvericin	CID 3010884	Antituberculous
49.	Enniatin B	CID 101925302	Antituberculous
50.	Enniatin B4	CID 164754	Antituberculous
51.	Enniatin G	CID 3010886	Antituberculous
52.	Oceanapia	CID 3010888	Antituberculous
53.	Psammaplysin A	CID 3010892	Antituberculous
54.	Oceanapiside	CID 44593641	Antituberculous
55.	1,3-Pyridinium Polymers	CID 9986729	Antituberculous
56.	[[5-(2-Amino-6-Oxo-1H-Purin-9-Yl)-3,4-Dihydroxy-Tetrahydrofuran-2-Yl]Methoxy-Hydroxy-Phosphoryl] Oxy	CID 84929	Antituberculous
57.	GDP-L-Galactose	CID 16072216	Antituberculous
58.	[[(2R,3S,4R,5R)-5-(2,4-Dioxopyrimidin-1-Yl)-3,4-Dihydroxy-Tetrahydrofuran-2-Yl]	CID 6857379	Antituberculous
59.	GDP-4-Keto-6-Deoxymannose	CID 644105	Antituberculous
60.	UDP-Xylose	CID 439446	Antituberculous
61.	Dtdp-4-Oxo-5-C-Methyl-L-Rhamnose;	CID 644105	Antituberculous
62.	Dtdp-4-Oxo-6-Deoxy-5-C-Methyl-L-Mannose	CID 439293	Antituberculous
63.	(2R,3S,4R,5R,6R)-3,4,5-Trihydroxy-6-[Hydroxy-[Hydroxy-	CID 443215	Antituberculous
64.	[[(2S,3R,5R)-3-Hydroxy-5-(5-Methyl-2,4-Dioxo-P	CID 11953944	Antituberculous
65.	Gdp-D-Rhamnose	CID 447152	Antituberculous
66.	GDP-D-Glycero-Alpha-D-Manno-Heptose	CID 439912	Antituberculous
67.	UDP-Galactopyranose (Natural Substrate Of UGM)	CID 21589156	Antituberculous
68.	1,4-Dihydroxy-2-Naphthoate Octaprenyltransferase	CID 18068	Antituberculous
69.	Aspartate-Β-Semialdehyde	CID 604249	Antituberculous
70.	Ursolic Acid	CID 5287708	Antituberculous
71.	Oleanolic Acid An	CID 64945	Antituberculous
72.	Tiliacorine	CID 10205	MDR TB
73.	2'- Nortiliacorinine	CID 124511658	MDR TB
74.	Tiliacorinine	CID 14527219	MDR TB
75.	Licarin B	CID 101670430	MDR TB, XDR TB, mono DR
76.	Eupomatenoid-7	CID 6441061	MDR TB, XDR TB, mono DR
77.	Dihydroguaiaretic Acid (Meso And (-) Forms)	CID 10314175	MDR TB, XDR TB, mono DR
78.	4-Epi-Larreatricin	CID 476856	MDR TB, XDR TB, mono DR
79.	5,4'-Dihydroxy-3,7,8,3'-Tetramethoxy Flavones	CID 11033399	MDR TB, XDR TB, mono DR
80.	2,4-Undecadienal	CID 5459184	MDR TB, XDR TB ,mono DR
81.	1α-Acetoxy-6β,9β-Dibenzoyloxydihydro-B-Agarofuran	CID 5367531	MDR TB, XDR TB, mono DR
82.	Leubethanol	CID 21593552	MDR TB, XDR TB,mono DR
83.	Abietane	CID 54669845	MDR TB, XDR TB, mono DR
84.	6,12-Dibenzoyl	CID 6857485	MDR TB, XDR TB, mono DR
85.	12-Methoxy Benzoyl	CID 76903	MDR TB, XDR TB, mono DR
86.	12-Chlorobenzoyl	CID 231963	MDR TB, XDR TB, mono DR
87.	12-Nitrobenzoyl Esters	CID 8501	MDR TB, XDR TB, mono DR
88.	Mono-Omethylcurcumin- Isoxazole	CID 7016100	MDR TB, XDR TB, mono DR
89.	Plumericin	CID 10249311	MDR TB, XDR TB, mono DR
90.	Isoplumericin	CID 5281545	MDR TB, XDR TB, mono DR
91.	Maritinone (Or) 3,3'- Biplumbagin	CID 5281543	MDR TB, XDR TB, mono DR
92.	Cis-Cinnamic Acid	CID 183757	MDR TB, XDR TB, mono DR
93.	Ethyl Pmethoxycinnamate	CID 5372954	MDR TB, XDR TB, mono DR
94.	Ursolic Acid	CID 5281783	MDR TB, XDR TB, mono DR
95.	Oleanolic Acid	CID 64945	MDR TB, XDR TB, mono DR
96.	Obtusifoliol	CID 10494	MDR TB, XDR TB, mono DR
97.	7,9-Dimethoxytariacuripyrone	CID 65252	MDR TB, XDR TB, mono DR
98.	Ent-1b,7a,14btriacetoxykaur-16-En-15-One	CID 96710	MDR TB, XDR TB, mono DR
99.	Plumbagin	CID 10205	MDR TB, XDR TB, mono DR
100.	Ambiguine	CID 10834980	MDR TB, XDR TB, mono DR
101.	Hapalindole H	CID 16109784	MDR TB, XDR TB, mono DR
102.	Hapalindole G	CID 21671525	MDR TB, XDR TB, mono DR
103.	Manilamine	CID 11067734	MDR TB, XDR TB, mono DR
104.	Nmethyl Angusilobine,	CID 101741721	MDR TB, XDR TB, mono DR
105.	19,20- (E) Vallesamine	CID 13891912	H37Rv
106.	20(S)-Tubotaiwine	CID 129317087	H37Rv
107.	6,7-Seco-Angustilobine	CID 13783720	H37Rv
108.	Globospiramine	CID 13891912	H37Rv
109.	5-Fluoro-3-Phenyl-1H-Indole	CID 53329268	H37Rv
110.	Indole-3-Carboxaldehyde 1,3,4-Thiadiazol-2- Yl-Hydrazone	CID 57345765	H37Rv
111.	Isoxazolo-	CID 11636795	H37Rv
112.	Mercaptopyrimido-	CID 20305010	H37Rv
113.	7-Hydroxymethylene-7, 8, 9, 10- Tetrahydrocyclohepta[B]Indol-6(5H)-Ones	CID 129781839	H37Rv
114.	Voacangine	CID 197060	H37Rv
115.	Hymenidin	CID 73255	H37Rv
116.	Monobromo Isophakellin	CID 6439099	H37Rv
117.	Ambroxol	CID 2442	H37Rv
118.	Denigrins A-C	CID 2132	H37Rv
119.	3-Methoxycarbonyl Carbazole	CID 231087	H37Rv
120.	Clauszoline J	CID 21252858	H37Rv
121.	2-Hydroxy-3-Formyl-7-Methoxy-Carbazole	CID 5315952	H37Rv
122.	Cryptolepine	CID 53324960	H37Rv
123.	Neocryptolepine	CID 82143	H37Rv
124.	Biscryptolepine	CID 390526	H37Rv
125.	(+)-8-Hydroxymanzamine A	CID 10457065	H37Rv
126.	(-)-Manzamine F	CID 5270765	H37Rv
127.	Manzamine A	CID 44445402	H37Rv
128.	6-Hydroxymanzamine E	CID 5468480	H37Rv
129.	Graveolinine	CID 826247	H37Rv
130.	Kokusagine	CID 11044132	H37Rv
131.	Bidebiline E (Dimericaporphine)	CID 5318829	H37Rv
132.	Liriodenine	CID 23642920	H37Rv
133.	Oxostephanine	CID 10144	H37Rv
134.	(-)-Nordicentrine	CID 343547	H37Rv
135.	Decarine [Or] Rutaceline	CID 10336429	H37Rv
136.	6-Acetonyldihydronitidine	CID 179640	H37Rv
137.	Nitidine	CID 10740045	H37Rv
138.	Chelirubine	CID 4501	H37Rv
139.	Macarpine	CID 161243	H37Rv
140.	Berberine	CID 440929	H37Rv
141.	Anonaine	CID 2353	
142.	Xylopine	CID 160597	MDR TB
143.	Anolobine	CID 160503	MDR TB
144.	Jatrorrhizine	CID 164710	MDR TB
145.	Sanguinarine	CID 72323	
146.	Chelerythrine	CID 5154	
147.	Vasicoline	CID 2703	H37Rv
148.	Vasicolinone	CID 626005	H37Rv
149.	Vasicinone	CID 627712	H37Rv
150.	Vasicine	CID 442935	H37Rv
151.	Adhatodine	CID 667496	H37Rv
152.	Anisotine	CID 5316460	H37Rv
153.	Vasicine Acetate	CID 442884	H37Rv
154.	Tryptanthrin	CID 11500	H37Rv
155.	Sarmentine	CID 73549	H37Rv
156.	Pyrrolidine	CID 6440616	H37Rv
157.	Sarmentosine	CID 31268	H37Rv
158.	Brachyamide B	CID 6438710	H37Rv
159.	Pellitorine	CID 14162526	H37Rv
160.	Brachystamide B	CID 5318516	H37Rv
161.	Malyngamide A	CID 14779548	H37Rv
162.	Malyngamide B	CID 14779548	H37Rv
163.	N-Isobutyl-(2E,4E)-2,4-Tetradecadienamide	CID 44246695	H37Rv
164.	1-Piperonyl Piperidine	CID 10731388	H37Rv
165.	Nummularine H	CID 21636624	H37Rv
166.	Mauritine M	CID 101204325	MDR TB
167.	Texalin	CID 53260757	MDR TB
168.	Malyngamide 4	CID 473253	MDR TB
169.	Malyngamide B	CID:5366244	MDR TB
170.	N-Isobutyl-(2E,4E)-2,4-Tetradecadienamide	CID 5785	MDR TB
171.	1-Piperonyl Piperidine	CID 442180	MDR TB
172.	Nummularine H	CID 2353	MDR TB
173.	Mauritine M	CID 637511	Antituberculous

**Table 3:** Docking results of Top ranked Phytochemicals interacting with WhiB6 (H37Rv)

Ligand	CID	MolDock Score	H-Bond	No of H- bonds	Interacting Amino Acid
UDP-galactopyranose	18068	-97.6778	-20.0687	9	Glu100, Arg101, Ser97, Arg96, Ala99, Pro105, Pyr104, Val106, Asp108
Methoxy-hydroxy-phosp-GDP-4-Dehydro-6-deoxy-D-mannose	439446	-105.492	-13.4574	10	Arg101, Ala99, Ser97, Glu100, Gly103, Tyr104, Pro105, Arg107, Asp108, Arg96
GDP-D-Rhamnose	439912	-111.961	-12.832	7	Asp108, Arg107, Val106, Pro105, Ala99, Glu100, Arg96
GDP-L-galactose	6857379	-115.809	-12.6431	11	Tyr104, Pro105, Arg107, Val106, Ala99, Glu100, Asp108, Arg96, Ser112, Leu92, Gly93
Oceanapia	3010892	-105.273	-11.5004	7	Gly103, Ala99, Glu100, Pro105, Arg96, Asp108, Arg107

**Table 4:** Docking results of MDR-TB first line drugs interacting with WhiB6 (H37Rv). Drugs shown in grey shade were found to be not interacting with WhiB6

Name	MolDockScore	H-Bond Score	No of H-Bond	Interacting Amino Acids
Pyrazinamide	-63.9854	-1.5602	4	Arg96, Val106
Isoniazid	-63.7479	0.554976	5	Asp108, Arg107,
				Arg96
Ethambutol	-78.1277	-7.05929	6	Asp108, Arg107,
				Val111
Streptomycin	34.2929	4.88673	No Interaction	
Rifampicin	967.454	-5.15092
